# Identification and binding-site mapping of RNA-binding proteins interacting with microRNA precursors in Arabidopsis

**DOI:** 10.1186/s12870-026-10297-z

**Published:** 2026-11-02

**Authors:** Andreas R. Winkel, Valentin W. Bitterer, Martin Lewinski, Marlene Reichel, Janina Lüders, Marion Scheibe, Maria Kalyna, Tom Laloum, Paula Duque, Dorothee Staiger, Falk Butter, Tino Köster

**Affiliations:** 1 RNA Biology and Molecular Physiology, Faculty of Biology, Bielefeld University, 33615 Bielefeld, Germany; 2 Present Address: Section for Computational Biology, Department of Biology, University of Copenhagen, Copenhagen, Denmark; 3 Institute of Molecular Biology (IMB), Ackermannweg 4, 55128 Mainz, Germany; 4 Friedrich-Löffler-Institut (FLI), Südufer 10, 17493 Greifswald, Germany; 5 Institute of Molecular Plant Biology, Department of Biotechnology and Food Science, University of Natural Resources and Life Sciences (BOKU), Vienna, 1190 Austria; 6 ISA – Sophia Agrobiotech Institute, Sophia Antipolis, 06903 France; 7 Université Côte d’Azur, Nice, France; 8 GIMM - Gulbenkian Institute for Molecular Medicine, Lisbon, Portugal; 9 Present Address: Plant Biotechnology, Faculty of Biology, Bielefeld University, 33615 Bielefeld, Germany

**Keywords:** MiRNAs, SR proteins, Arabidopsis thaliana, Pri-miRNA processing, RNA pulldown, RNA-binding protein, ICLIP

## Abstract

**Background:**

MicroRNAs (miRNAs) play key roles in modulating gene expression. Upon transcription, primary miRNA transcripts (pri-miRNAs or *MIRNAs*) fold into stem-loop structures. Endonucleolytic cleavage releases a miRNA/miRNA* duplex from the stem, which is subsequently matured. In higher plants, the pri-miRNA hairpins vary widely in length and structure. Pri-miRNA processing is extensively regulated by RNA-binding proteins (RBPs) and the range of underlying mechanisms continues to expand.

**Results:**

Here, we performed an unbiased screen to identify *Arabidopisis thaliana* candidate RBPs that interact with pri-miRNAs, using *pri-miRNA159a* and *pri-miRNA398b* as paradigms. Nucleoplasmic proteins were significantly enriched in pulldowns with bead-immobilized in vitro transcripts of the stem-loop regions of these pri-miRNAs compared with pulldowns using empty beads. The enriched proteins included several serine/arginine-rich (SR) splicing factors, notably RS31 and SR34a, extending previous findings that RS40 and RS41 bind pri-miRNAs and participate in miRNA biogenesis. Mutant analysis indicated that selected candidate interactors affect pri-miRNA and/or mature miRNA accumulation. To validate candidate interactions with pri-miRNAs in vivo, we performed individual-nucleotide resolution crosslinking and immunoprecipitation (iCLIP). Whereas our conventional plant iCLIP procedure detected only a few crosslink sites on pri-miRNAs, likely because of the low abundance of these transcripts, our improved plant iCLIP2 protocol enabled the identification of binding sites on numerous pri-miRNAs. RS31 and SR34a bound overlapping sets of pri-miRNAs and contacted both stem-loop regions and flanking regions upstream and downstream of the stem-loop. Binding-site mapping resolved overlapping and distinct contact regions and highlighted the importance of complete pri-miRNA annotations for interpreting interactions beyond annotated transcript boundaries.

**Conclusion:**

In vitro pulldowns of nucleoplasmic proteins identified candidate RBPs associated with pri-miRNA stem-loops, and the improved plant iCLIP2 protocol revealed in vivo binding of RS31 and SR34a to a subset of pri-miRNAs. Together, these findings expand the repertoire of candidate RBPs implicated in plant miRNA biogenesis and support a role for SR proteins as candidate regulators of miRNA abundance.

**Supplementary Information:**

The online version contains supplementary material available at https://doi.org/10.1186/s12870-026-10297-z.

## Background

MicroRNAs (miRNAs) are noncoding RNAs with a length of 20–24 nucleotides (nt) that regulate the expression of cognate target genes by eliciting mRNA cleavage or translational inhibition [1, 2]. MiRNAs are generated from RNA polymerase II transcripts with internal stem-loop structures, the primary miRNAs (pri-miRNAs). A first cleavage by an RNase III protein releases the stem-loop (precursor miRNA, pre-miRNA) which is further processed to the miRNA/miRNA* duplex. After methylation of the 3´ ends by HUA ENHANCER1 (HEN1), the mature miRNA is loaded into ARGONAUTE 1 (AGO1) while the miRNA* strand is mostly degraded [3–5]. In higher plants, the endonucleolytic cleavages are performed mainly by the RNase III type enzyme DICER-LIKE1 (DCL1) [6]. Along with DCL1, the double-stranded RNA-binding protein (RBP) HYPONASTIC LEAVES 1 (HYL1) and the zinc finger protein SERRATE (SE), collectively designated as the microprocessor complex, are required to ensure efficient and accurate processing [7–9]. More recently, it was found that phase separation of SE promotes pri-miRNA processing by the microprocessor complex [10]. Apart from the microprocessor complex, a suite of proteins affects pri-miRNA processing. DAWDLE (DDL) and PLEIOTROPIC REGULATORY LOCUS 1 (PRL1) stabilize pri-miRNAs and promote miRNA biogenesis [11, 12]. Additional members of the MOS4-ASSOCIATED COMPLEX interact with and promote the activity of DCL1 [12–15]. The Prp28-related DEAD-box protein SMALL1 (SMA1) associates with the DCL1 processing machinery and supports efficient conversion of pri-miRNA substrates [16]. TOUGH (TGH) interacts with pri-miRNAs and promotes their interaction with HYL1 [17].

It is increasingly becoming clear that the secondary structure of the pri-miRNA stem-loop plays a critical role in processing. For example, CHROMATIN REMODELLING 2 (CHR2), the ATPase subunit of the SWI/SNF chromatin remodelling complex, displays RNA helicase activity to unwind the pri-miRNA stem-loops, leading to reduced levels of mature miRNAs [18].

Pri-miRNAs contain a lower stem and flanking single-stranded basal segments below the double-stranded region corresponding to the mature miRNA and its complementary miRNA*. Above the duplex region, there are an upper stem and the terminal loop. The initial cleavage by DCL1 occurs 15–17 nt away from the ssRNA-dsRNA junction. Due to extensive heterogeneity in length and structure of pri-miRNA stem-loops, different modes of plant pri-miRNA processing have been distinguished based on the direction and number of cuts in higher plants [19]. Accordingly, pri-miRNAs can be processed from base-to-loop or from loop-to-base [19, 20]. In the “short base-to-loop” processing mode (e.g., pri-miR172 and pri-miR398 families), an imperfectly paired lower stem of ∼15 nt below the miRNA/miRNA* duplex followed by an internal loop determines the site of the initial pri-miRNA cleavage [21]. Subsequent cleavage of the pre-miRNA at a fixed distance of ∼21 nt from the first cut then releases the miRNA/miRNA* duplex. Two pri-miRNA families, pri-miR156 and pri-miR160, are processed by a “short loop-to-base” mechanism where the first cut is guided by an upper stem segment and a small loop.

Besides, further modes of processing exist in which two additional cuts are required to release the mature miRNA (sequential base-to-loop or sequential loop-to-base) [19, 20]. These successive cuts along the stem allow for generation of additional low abundant small RNAs. In the pri-miR159 and pri-miR319 families with their long stem segments the first cut occurs near the loop and three more sequential cuts follow to release the mature miRNA in this “sequential loop-to-base” mechanism.

Transcription of pri-miRNA genes widely responds to endogenous cues and environmental stimuli including abiotic and biotic stress [22]. Processing of pri-miRNAs occurs co-transcriptionally once the secondary structure stem-loop is folded [23]. While for pri-miRNAs processed from loop-to-base all steps occur co-transcriptionally, for pri-miRNAs processed from base-to-loop only the first cleavage is executed co-transcriptionally, while further processing occurs on pri-miRNAs released into the nucleoplasm [23]. Notably, some pri-miRNAs can be processed both co-transcriptionally and post-transcriptionally, depending on environmental conditions. Thus, plants exquisitely fine-tune processing of miRNA precursors into mature miRNAs to adjust the miRNome. Accordingly, numerous RBPs have been implicated in the regulation of pri-miRNA processing.

Interaction of several RBPs with pri-miRNAs has been demonstrated using RNA immunoprecipitation [24, 25]. Among those, *Arabidopsis thaliana* GLYCINE-RICH RNA-BINDING PROTEIN 7 binds to selected pri-miRNAs in vivo to impact their processing [24]. RS40 and RS41 were the first plant SR proteins implicated in miRNA biogenesis in plants, supporting proper miRNA accumulation and strand selection. They interact with HYL1 and SE, bind both intron-containing and intronless pri-miRNAs in vivo, and contribute to the splicing of selected pri-miRNAs, supporting functions in miRNA biogenesis beyond splicing [25].

So far, the regions within the precursor that interact with the RBPs remain unknown. Ultraviolet (UV) crosslinking based techniques such as individual-nucleotide resolution crosslinking and immunoprecipitation (iCLIP) determine in vivo binding sites of RBPs with single nucleotide resolution [26]. Irradiation with 254-nm UV light is employed to covalently link RNAs and bound proteins to preserve the native interactions. RNA-protein complexes are recovered from lysates by immunoprecipitation and the associated RNAs are radioactively labelled. After purification of the RNA-protein complexes by denaturing gel electrophoresis the region above the expected molecular weight, containing protein with associated RNAs, is processed for RNA isolation to generate sequencing libraries. During reverse transcription, the remnants of the crosslinked protein stall the reverse transcriptase, causing cDNAs to terminate immediately before the crosslink site. Sequencing these cDNAs enables determination of binding sites at single-nucleotide resolution. Although UV crosslinking-based methods were initially developed for mammalian cell monolayers or translucent tissues, iCLIP has recently been adapted for plants, whose multicellular, opaque tissues and rigid cell walls complicate crosslinking and recovery [27, 28].

In the conventional iCLIP procedure, one bipartite linker is used to prime cDNA synthesis, and the attachment of the linker to the cDNA 3´ end is accomplished *via* intramolecular circularization and relinearization. In iCLIP2, a more efficient ligation of the second linker directly to the cDNA 3´ end is used, as done in the infrared CLIP protocol [29]. Furthermore, the protocol includes a pre-amplification step prior to size selection of the cDNAs and uses bead-based instead of gel-based size selection and employs beads instead of purification by repeated ethanol precipitation that was introduced in eCLIP to avoid sample loss [30]. Recently, the updated iCLIP2 protocol which provides increased sensitivity has been implemented in plants [31–34].

To systematically identify candidate RBPs that may impact pri-miRNA processing, we have established RNA affinity pulldown with in vitro transcribed pri-miRNA stem-loops incubated with protein extracts from plant nuclei. Bound proteins were identified by mass spectrometry and categorized according to enrichment relative to controls. Binding of the candidate RBPs to the miRNA stem-loops in vivo was validated using plant iCLIP2. Mutants deficient in selected interacting candidate RBPs were monitored for alterations in miRNA biogenesis, employing small RNA northern blot hybridization.

## Methods

### Cloning and in vitro transcription of pri-miRNA stem-loops

A synthetic gBlock™ (IDT, Coralville, IA, USA) encoding the *MIR398b* hairpin together with an upstream T7 promoter was inserted into pUC19 between the *Eco*RI and *Xba*I sites (Additional file 1, Table S1). For in vitro transcription of *MIR398b*-SL, the plasmid pUC19-T7-MIR398b-SL containing the *MIR398b* stem-loop region downstream of the T7 promoter was linearized with *Xba*I and gel purified using the NucleoSpin^®^ Gel and PCR Clean up kit (Macherey-Nagel, Düren, Germany). A second gBlock™ (IDT, Coralville, IA, USA), comprising the annotated *MIR159a* hairpin, the T7 promoter, a 20-nt Csy4 hairpin, and spacer sequences, was cloned into pUC19 using *Eco*RI and *Hind*III (Additional file 1, Table S1) [35]. To omit in vitro transcription of the Csy4 hairpin, the stem-loop region of *MIR159a*-SL was PCR-amplified from the plasmid pUC19-T7-Csy4-MIR159a-SL using primers T7-MIR159a-SL_for and MIR159a-SL_rev. The templates for in vitro transcription were purified via the NucleoSpin^®^ Gel and PCR Clean up kit (Macherey-Nagel, Düren, Germany). Biotin-labelled RNA was generated with the MEGA shortscript™ T7 kit (Invitrogen, Carlsbad, CA, USA) by adding biotinylated dUTP in accordance with the supplier’s protocol. Following transcription, the RNA products were cleaned with the RNA Clean & Concentrator-25 system (Zymo Research, Freiburg, Germany). RNAfold was used to computationally model the predicted folds of *MIR159a-SL* and *MIR398b-SL* [36].

### Plant material

All lines used in this study are in the *Arabidopsis thaliana* Col-0 ecotype. The following mutant and transgenic lines have been described previously: *rs31-1* (SALK_021332; At3g61860) [37, 38], 35 S::RS31 overexpressing the RS31 coding region (At3g61860) under control of the Cauliflower Mosaic Virus (CaMV) 35 S promoter (RS31-ox) [38], RS31::RS31-GFP, expressing RS31 fused to GFP under the control of the endogenous promoter in the *rs31-1* mutant background [38], *rs31a* line carrying an artificial miRNA against *RS31a* (amiR-31a-E2; At2g46610) [39], *sr34a-1* (GK-803A10; AT3G49430) [34], SR34a::SR34a-GFP, expressing SR34a fused to GFP under the control of the endogenous promoter in the *sr34a-1* mutant background, *sr34-1* mutant (SALK_106067; At1g02840) [40], *sr45-1* (SALK_004132; AT1G16610) [41], *brr2a-2* (*cäö*) carrying a G to A transition in the At1g20960 gene that results in a T895I missense mutation [42], *nuc1-2* (SALK_002764; At1g48920) [43], and *rs40 rs41* double mutant (WiscDsLox382G12 x SAIL_64_C03; AT4G25500, AT5G52040) [44]. Transgenic plants expressing GFP alone under the control of the CaMV 35 S promoter have also been described previously [38].

Following surface sterilization, Arabidopsis seeds were plated on half-strength Murashige and Skoog (MS) medium (Duchefa, Haarlem, Netherlands) [45]. Cultivation took place in Percival growth chambers (CLF, Wertingen, Germany) at 20 °C, with 12 h of light alternating with 12 h of darkness. At dusk, aerial material was collected from 14-day-old Col-0 plants for nucleoplasmic extract preparation, RNA measurements, and iCLIP.

For salt and ABA treatments, 12-day-old Arabidopsis plants grown on half-strength MS medium were transferred to half-strength MS plates supplemented with either 200 mM NaCl or 100 µM abscisic acid (ABA) and incubated under the same defined growing conditions for 48 h before harvest.

### Preparation of nucleoplasmic protein extracts

A 5 g portion of Arabidopsis Col-0 wild-type tissue was pulverized under liquid N2 and mixed with 5 mL nLB containing 20 mM Tris-HCl (pH 7.4), 25% glycerol, 20 mM KCl, 2 mM Na_2_-EDTA (pH 8.0), 2.5 mM MgCl_2_, 250 mM sucrose, and 5 mM dithiothreitol (DTT). Unless stated otherwise, the procedure was carried out on ice or at 4 °C. The homogenate was passed first through a 100-µm nylon mesh and then through two Miracloth layers (22–25 μm; Calbiochem). The filtered homogenate was centrifuged for 30 min at 1500 x g. The supernatant, representing the cytosolic fraction, was collected in a new tube. For the nuclear wash, the pellet was dispersed in 12 mL NRBT (20 mM Tris-HCl, pH 7.4, 25% glycerol, 2.5 mM MgCl2, and 0.2% Triton X-100) and centrifuged for 15 min at 1500 x g. The NRBT wash was conducted four or five times, until the supernatant was clear. Nucleoplasmic proteins were then extracted by taking up the final pellet in three pellet volumes (approximately 600 µL) of PEB+ containing 20 mM HEPES-KOH (pH 7.5), 5% glycerol, 1.5 mM MgCl_2_, 0.2 mM Na_2_-EDTA (pH 8.0), 420 mM NaCl, 5 mM DTT, and 1× cOmplete protease inhibitor (Roche, San Francisco, CA, USA), as reported previously [46]. Before the pulldown assays, the NaCl concentration of the extract was adjusted to 150 mM. Total protein was quantified with the QubitTM Protein Assay Kit (Thermo Fisher Scientific).

### Immunoblot

Total protein, cytoplasmic, and nucleoplasmic samples were examined by immunoblotting according to the procedure described previously [27]. The primary antibody panel comprised rabbit polyclonal antibodies against HISTONE 3 (Agrisera, Vännäs, Sweden, AS10710; 1:5000), UDP-GLUCOSE PYROPHOSPHORYLASE (Agrisera, AS11 1739; 1:5000), and SERRATE (Agrisera, AS09532A; 1:1000). For secondary detection, HRP-conjugated anti-rabbit IgG (Sigma-Aldrich, Burlington, MA, USA, A0545) was applied at 1:2500. Chemiluminescent signals were recorded on either a Stella imager (Raytest) or a Fusion-FX6 instrument (Vilber, Germany).

### Biotin-based RNA pulldown

For RNA refolding, 3.0 µg of biotinylated, in vitro transcribed *pri-miR398b* RNA was diluted in 250 µL of RNA binding buffer (20 mM Tris-HCl, pH 7.5, 100 mM NaCl, and 3 mM MgCl₂). The RNA was heated at 70 °C for 10 min, cooled on ice for 2 min, and subsequently incubated at 20 °C for 10 min. The refolded RNA was combined with 75 µL of Dynabeads MyONE Streptavidin C1 beads (Thermo Scientific) and incubated at 4 °C for 90 min with continuous shaking. Finally, the beads were washed three times with 150 µL of RNA binding buffer per wash.

Beads loaded with the in vitro transcripts or empty beads as control were incubated with 2 mL of nucleoplasmic proteins (800–1100 µg) in PEB+ containing 20 mM HEPES-KOH (pH 7.5), 5% glycerol, 1.5 mM MgCl_2_, 0.2 mM Na_2_-EDTA (pH 8.0), 150 mM NaCl, 5 mM DTT, and 1× cOmplete protease inhibitor (Roche, San Francisco, CA, USA) for 90 min at 4 °C. After three washes in 500 µL protein washing buffer (20 mM Tris-HCl, pH 7.5, 100 mM NaCl, 5% glycerol, 1mM TCEP, 0.1% Triton X-100) beads were either processed for quality control or for mass spectrometry. For RNA analysis, 5% of the slurry (beads after pulldown, BAP) was heated for 5 min at 95 °C in formamide containing loading buffer (90% formamide, 9.8% glycerol, 0.1% bromphenol blue, 0.1% xylene cyanol). For protein analyses, 95% of the slurry was resuspended in 40 µL SDS sample buffer and subjected to SDS-PAGE. For either silver staining or mass spectrometry, the slurry of samples (E+) and the empty beads (E-) were resuspended in 40 µL LDS sample buffer (Novagen™ NuPAGE™ LDS sample buffer, Thermo Scientific) and incubated for 10 min at 70 °C.

### Silver staining

Proteins were resolved at 100 V in 1x MOPS buffer on 4–12% NuPAGE™ Novex Bis-Tris precast gels (Thermo Scientific), after which silver staining was carried out as reported previously [47].

### Mass spectrometry and data analysis

For the short preparative separation, proteins were run for 8 min at 180 V in MES buffer on 4–12% NuPAGE™ Novex Bis-Tris precast gels (Thermo). The gels were fixed, stained with Coomassie Brilliant Blue (Carl Roth, Karlsruhe, Germany), cut into small pieces, washed with 50% ethanol/25 mM ammonium bicarbonate, and dehydrated in neat acetonitrile. Reduction was carried out for 1 h at 56 °C in 10 mM DTT/50 mM ammonium bicarbonate (Sigma-Aldrich), followed by alkylation for 45 min in darkness at room temperature with 50 mM iodoacetamide in 50 mM ammonium bicarbonate (Sigma-Aldrich). After another dehydration with acetonitrile (VWR), the gel fragments were digested overnight at 37 °C with 1 µg MS-grade trypsin (Serva). Peptides were recovered by two successive extractions with 30% acetonitrile and two with 100% acetonitrile; the pooled extracts were reduced to approximately 100 µL in an Eppendorf concentrator. StageTips were used for desalting [48] and chromatographic separation was performed on a New Objective nanocapillary packed in-house with Reprosil C18 (Dr. Maisch GmbH, Ammerbuch, Germany). This column was coupled to an Easy nLC 1000 (Thermo) and operated for 90 min with an acetonitrile gradient from 5% to 60% in 0.1% formic acid at 225 nL/min. Electrospray was introduced through the nanospray source of a Q Exactive Plus mass spectrometer (Thermo). Data-dependent acquisition used HCD and selected the ten most intense precursors for MS/MS after each Orbitrap full scan.

MaxQuant 1.5.2.8 [49] was used to analyze the raw spectra against the TAIR10 proteome comprising 35,386 entries. Processing otherwise followed the default MaxQuant configuration, with label-free quantification enabled [50], FastLFQ disabled, and matching between runs switched on. Contaminant records, reverse-database matches, and proteins identified solely by a modification site were excluded before statistical evaluation. For absent intensities, values near the quantification limit were sampled from a compressed beta distribution shifted towards lower abundance. For each protein, mean enrichment across the four replicates was calculated, and replicate variation was evaluated in R with Welch’s t-test. Significance was assigned to proteins meeting both thresholds (log_2_ fold change ≥ 1.0 and *p* ≤ 0.05); enrichment and p values were displayed after logarithmic transformation.

### Analysis of GO terms and protein domains

Overrepresentation of GO categories and protein domains was evaluated with ThaleMine, applying its default background set and a Holm-Bonferroni adjustment [51].

### Small RNA gel blots

Each biological replicate consisted of pooled material from at least ten plants, from which total RNA was extracted with TRIzol^®^ reagent (Invitrogen). For small-RNA blotting, 5 µg total RNA was separated in Tris-borate-EDTA buffer on a 17% polyacrylamide gel containing 8 M urea, transferred by capillary action to a GeneScreen membrane, and UV-crosslinked at 254 nm with 120 mJ/cm^2^. The membrane was incubated with 5′-biotinylated antisense DNA probes (Metabion, Planegg, Germany) listed in Additional file 1, Table S2, using 7% SDS, 200 mM Na_2_HPO_4_ (pH 7.0), and 5 µg/mL salmon sperm DNA as the hybridization solution. Probe hybridization proceeded for 12–15 h at 38 °C under end-over-end mixing. Membranes were then washed twice for 15 min at 38 °C in 1x SSC containing 0.1% SDS. Bound biotinylated oligonucleotides were visualized with the Nucleic Acid Detection Module Kit (Thermo Fisher Scientific) following the supplied protocol. Chemiluminescent images were acquired with a Stella (Raytest) or Fusion-FX6 (Vilber, Germany) system, and band intensities were measured in ImageJ version 1.53 (W.S. Rasband, NIH, Bethesda, MD, USA) [95]. Differences were tested with Student’s t-test, using *p* ≤ 0.05, *p* ≤ 0.01, and *p* ≤ 0.001 as the thresholds indicated by one, two, and three asterisks, respectively.

### RT-qPCR

RNA destined for RT-qPCR was converted to cDNA with AMV reverse transcriptase (EURX Roboklon, Berlin, Germany). Reactions of 10 µL containing iTaq SYBR GREEN supermix (BioRad, Hercules, CA, USA) were run on a CFX96 instrument (BioRad) for 45 cycles, each comprising 15 s at 95 °C and 30 s at 60 °C. Relative transcript abundance was derived from Cq measurements while accounting for the individual amplification efficiency of each primer pair [52]. PP2A (At1g13320) served as the reference transcript; values are reported as the mean of independent biological replicates with their standard deviation, unless a figure legend specifies another presentation. Differences were evaluated with Student’s t-test, with significance thresholds of *** *p* ≤ 0.001, ** *p* ≤ 0.01, and * *p* ≤ 0.05.

### Plant iCLIP2

RS31::RS31-GFP, SR34a::SR34a-GFP and 35 S::GFP plants were subjected to irradiation with 254 nm UV light (2000 mJ cm^-2^) at a constant temperature of 20 °C and above-ground tissues were subsequently flash frozen. For each replicate, three grams of plant material were processed, as previously described for plant iCLIP2 [31, 32]. After sequencing, raw reads were processed following the bioinformatics workflow described by Lewinski et al. (“Part 2. bioinformatic analysis”) [32]. Briefly, uniquely mapped reads were combined across biological replicates after removal of PCR duplicates, and PureCLIP was used to identify enriched crosslink positions. Binding sites were defined as 3-nucleotide regions centred on the strongest peak within each cluster of adjacent peaks. Sites supported by crosslinks at only one nucleotide position were excluded. BindingSiteFinder removed the lowest-scoring 2% of peaks globally and reproducible binding sites were required to have crosslink counts above the 30th-percentile threshold within each gene in at least two of three biological replicates for RS31::RS31-GFP and both replicates for SR34a::SR34a-GFP. Reproducibility was not assessed for the GFP control because too few uniquely mapped reads were available.

### MIR annotation file

Genomic pri-miRNA coordinates annotated in mirEX^2^ [53] were combined with those reported by Lepe-Soltero et al. (Additional file 2, Table S4) [54]. For each *MIRNA*, the annotation covering the longest genomic interval was selected and converted into BED and GTF formats. The annotation source was indicated in the name field using miRXXX_M2 for mirEX^2^ and miRXXX_LS for Lepe-Soltero. *MIRNAs* present in only one source were labelled oM2 (only mirEX^2^) or oLS (only Lepe-Soltero).

### Motif discovery

The sequence consisting of nine nucleotides at each binding locus was extracted utilizing bedtools getfasta in a strand-specific configuration [55] in conjunction with the TAIR10 genome. The process of motif discovery was divided into two distinct methodologies. The initial methodology employed Expectation-Maximization via STREME 5.4.1 [56] to ascertain significant motifs, while the subsequent methodology focused on k-mer distributions surrounding the designated peak position within the binding site region. Both methodologies utilized an identical set of sequences (for the purpose of motif discovery).

### Differential gene expression

Mapped reads in the BAM format were obtained from previous paired-end RNA-sequencing experiments encompassing three biological replicates each of *A. thaliana* wild-type (Col-0), the *rs31-1* loss-of-function mutant, and the RS31-ox line [38]. Transcript quantification was performed using StringTie (v2.2.1), with a reference GTF file containing pri-miRNA annotations (GFF2 format). Quantification was executed using the following parameters: stringtie Sample.bam -p 4 -G primiRNA.gtf -e -b Sample/. Gene-level FPKM (Fragments Per Kilobase of transcript per Million mapped reads) values were extracted from the resulting t_data.ctab files for each replicate and aggregated using a custom R script. Differential expression between genotypes was assessed by performing two-sample t-tests on FPKM values, comparing wild-type with both the mutant and overexpressor lines.

## Results

### Identification of candidate pri-miRNA stem-loop interacting proteins via biotin-based pulldown

**Fig. 1:**
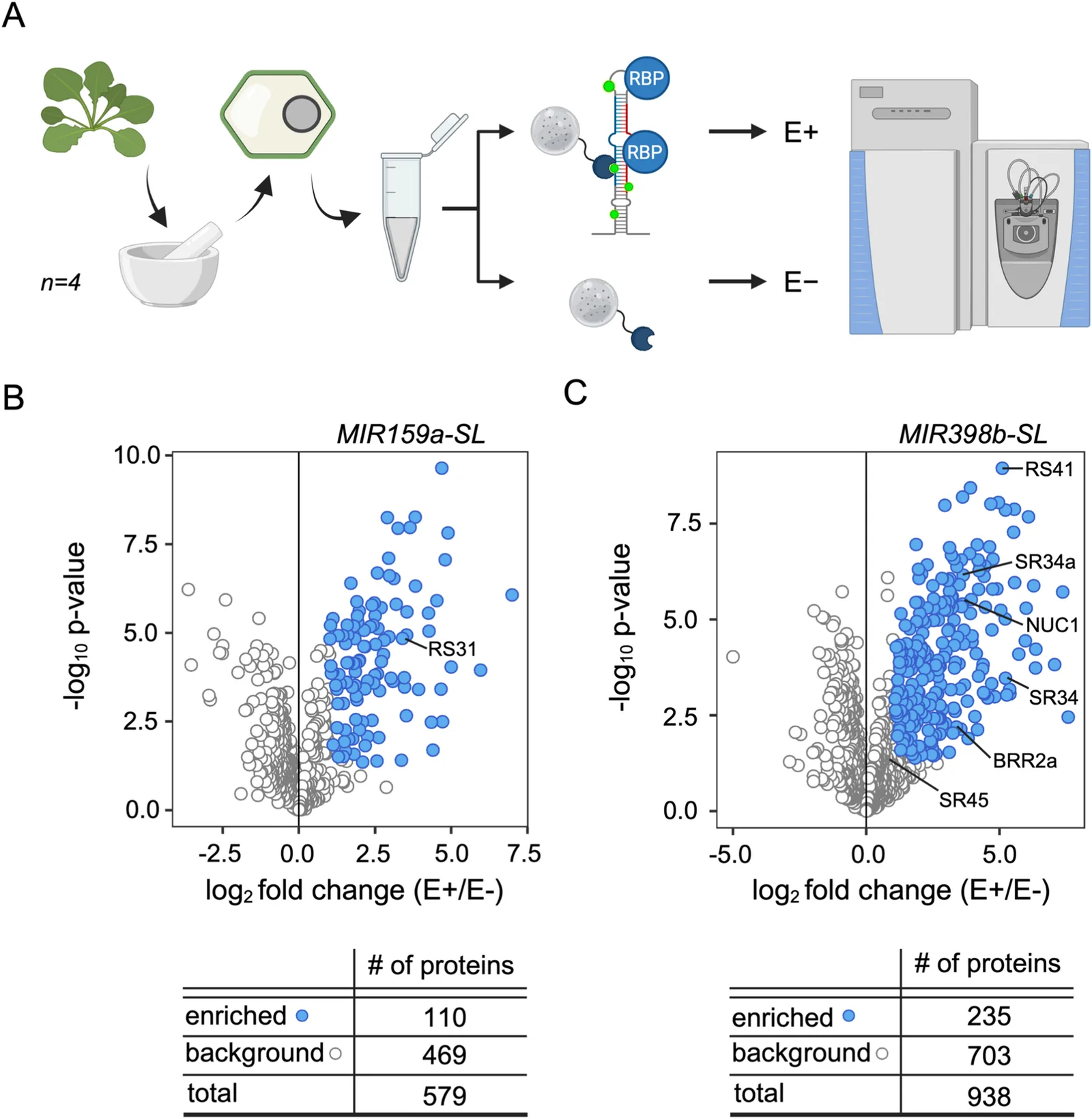
Biotin-based pulldown to identify candidate proteins interacting with *MIRNA *stem-loops. **A** Experimental strategy: Nucleoplasmic protein extracts were prepared from four independent batches of Arabidopsis plants and incubated with biotinylated *MIRNA* stem-loop transcripts immobilized on streptavidin beads. Eluted proteins (E+) were identified by mass spectrometry and enrichment was determined relative to eluates from empty beads (E-) (Welch *t*-test, *n* = 4). **B-C** Volcano plots show proteins recovered with the *MIR159a-SL *(**B**) and the *MIR398b-SL *(**C**). Proteins with a log_2_ fold change > 1 and a p-value ≤ 0.05 (Welch *t*-test, *n* = 4) are defined as candidate interactors, whereas all other proteins are considered background. Proteins selected for further analysis are indicated.

A growing number of RBPs have been implicated in the complex and dynamic regulation of plant miRNA biogenesis yet understanding the specific regulatory proteins that interact with distinct miRNA precursors remains insufficiently explored. To identify candidate RBPs regulating the processing of miRNA precursors in Arabidopsis, we developed an RNA affinity pulldown based on biotinylated in vitro transcripts of pri-miRNA (*MIRNA*) stem-loops (*SL*). The experimental strategy is outlined in Fig. 1A. We chose the *SL* regions of two pri-miRNAs that are processed from loop-to-base and from base-to-loop, respectively. *Pri-miR159a* is processed from loop-to base in a sequential manner to release the mature miR159 [57, 58]. The template for in vitro transcription comprised 198 nucleotides downstream of the T7 RNA polymerase promoter (Additional file 1, Table S1, Additional file 3, Fig. S1A). In contrast, *pri-miR398b* is processed in base-to-loop direction to release the mature miR398. The 134-nt *MIR398b-SL* bait was designed to retain the approximately 15-nt basal stem that positions the first DCL1 cut, thereby preserving the structural elements required for correct processing (Additional file 1, Table S1, Additional file 3, Fig. S1B). For RNA affinity pulldown, the biotinylated *MIR159a-SL* and *MIR398b-SL in vitro* transcripts were immobilized on streptavidin beads.

As pri-miRNA processing occurs entirely in the nucleus in higher plants, both *MIR159a*-*SL* and *MIR398b-SL *in vitro transcripts were incubated with nuclear extracts to identify candidate interactors. Therefore, nuclei were isolated, and extracts enriched in nucleoplasmic proteins were prepared without lysing nuclei, as outlined in Additional file 3, Fig. S2A. The enrichment of nucleoplasmic proteins and the purity of the extracts was demonstrated by immunoblots using compartment-specific antibodies (Additional file 3, Fig. S2B) (Additional file 4, Fig. S18: Uncropped blots to Fig. S2B).

The bead immobilized in vitro transcripts of *MIR159a-SL* and *MIR398b-SL*, respectively, were then incubated with the nucleoplasmic proteins. Denaturing gel electrophoresis before the pulldown (input) and after the pulldown (supernatant and eluate) confirmed the integrity of the RNAs during the incubation (Additional file 3, Fig. S3A, B) (Additional file 4, Fig. S19: Uncropped gels to Fig. S2A, B). After extensive washing of the beads, bound proteins were heat-eluted in LDS sample buffer. Comparison of the proteins eluted from beads loaded with either *MIR159ab-SL* or *MIR398b-SL* (E+) and proteins eluted from empty beads (E-) by SDS-PAGE followed by silver staining confirmed the co-precipitation of proteins interacting with the *MIRNA-SLs* (Additional file 3, Fig S3C, D) (Additional file 4, Fig. S19: Uncropped gels to Fig. S2C, D).

The remainder of the eluates were subjected to MS in four biological replicates. Candidates were designated as interactors when their abundance over the empty-bead control reached a log_2_ fold change of at least 1.0 together with *p* ≤ 0.05 (Fig. 1B, C) (Additional file 5, Table S5 and S6). Proteins not meeting these criteria were not considered further as significantly enriched candidates. In the four preparative pulldowns for the *MIR159a-SL*, we retrieved 579 proteins. 110 of these were significantly enriched relative to proteins retrieved with empty beads (E-) (Fig. 1B, Additional file 5, Table S5). For the *MIR398b* stem-loop, 938 proteins were retrieved in total with 235 being significantly enriched (Fig. 1C, Additional file 5, Table S6).

### **Categorization of the proteins interacting with the*MIRNA*stem-loops**

**Fig. 2:**
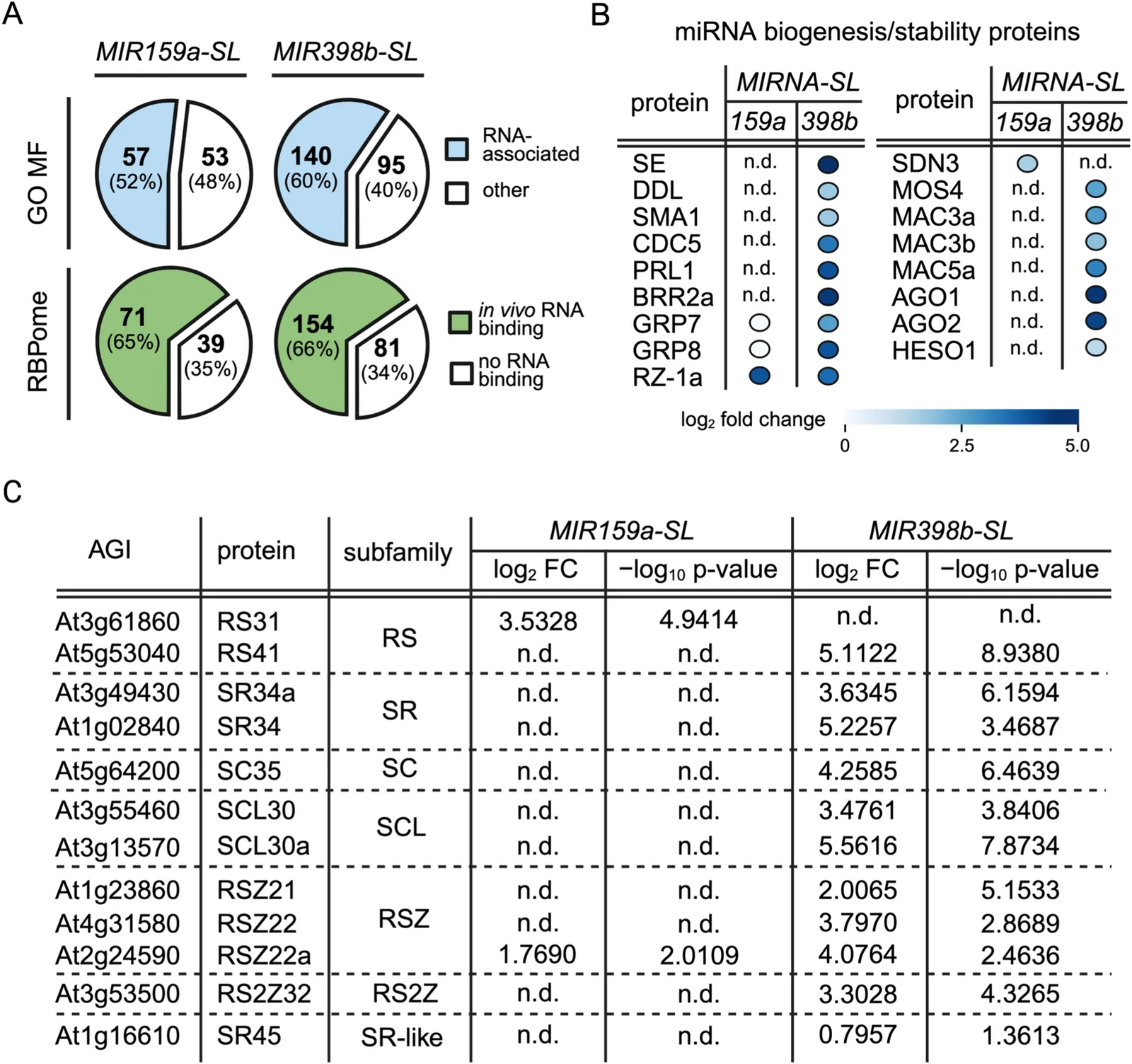
Functional classification and properties of candidate *MIRNA-SL* interactors. **A** Pie charts displaying the proportion of enriched proteins that are associated with RNA-binding-related molecular function (MF) Gene Ontology (GO) terms (top) and overlap with the Arabidopsis RNA-binding proteomes (RBPomes) determined by RNA interactome capture (bottom) for *MIR159a-SL* interactors and *MIR398b-SL* interactors. **B** Proteins associated with *MIRNA* processing/stability enriched in the pulldowns with *MIR159a-SL* and *MIR398b-SL*. **C** SR proteins identified as *MIR159a-SL* or *MIR398b-SL* interactors. SR-like protein SR45 was detected but did not meet the fold-change cut-off. n.d. – not detected.

To functionally categorize the proteins enriched in the pulldown assays, we conducted a systematic gene ontology (GO) term analysis of the interactors’ molecular identities. Among the candidate interactors of *MIR159a-SL* and *MIR398b-SL*, 52% and 60%, respectively, were annotated with RNA-associated molecular function GO terms (Fig. 2A, top). Accordingly, the interactors were also enriched for RNA-associated GO terms in Biological Process (BP) (Additional file 6, Table S7 and S8). This is consistent with potential functions as regulators of miRNA biogenesis. Importantly, two thirds of the interactors were present among the RNA binding proteomes (RBPome) in Arabidopsis determined by RNA interactome capture using oligo(dT) pull-down or purification based on phase extraction (Fig. 2A, bottom, Additional file 7, Table S9) [47, 59–64]. This provides independent support for RNA-binding activity of many of the identified proteins in vivo.

Furthermore, among the protein domains enriched in the interactors characterized RNA-binding and nucleotide-binding domains are predominantly found (Additional file 8, Table S10 and S11).

To further validate our purification strategy, we asked whether the eluates contained established components of the miRNA-biogenesis pathway (Fig. 2B). For *MIR159a-SL*, no components of the miRNA core processing complex (defined as DCL1, HYL1, SE) were found. However, among the interactors were RZ-1a and SDN, proteins that have already been associated with miRNA biogenesis and stability, respectively [35, 65]. Additionally, the glycine-rich RNA binding proteins GRP7 and GRP8, for which direct in vivo binding to pri-*miR159a* has already been confirmed, are also among the identified, albeit not significantly enriched proteins [24].

For *MIR398b-SL*, SE of the core processing complex, AGO1, AGO2, and numerous proteins associated with pri-miRNA processing were significantly enriched (Fig. 2B). In particular, the BRR2a RNA helicase, a component of the U5 small nuclear ribonucleoprotein (U5 nRNP), recently shown to interact with pri-miRNAs, ensuring proper folding for processing was among the interactors [66]. Additionally, another component of U5 snRNP, GAMETOPHYTIC FACTOR 1 (GFA1, At1g06220), was enriched (Additional file 5, Table S6). Notably, we identified several SR proteins. Although RS40 and RS41 have been implicated in pri-miRNA processing, the impact of other SR and SR-like proteins in pri-miRNA processing is not well understood (Fig.2C). The SR protein family in plants is expanded compared to mammals. Arabidopsis harbors 18 SR proteins classified into six subfamilies and two SR-like proteins, SR45 and SR45a [67]. Pulldown of *MIR159a-SL* led to enrichment of RS31, a member of the plant-specific RS subfamily characterized by two RRMs (RS31, RS31a, RS40, and RS41). Furthermore, RSZ22a (RSZ subfamily with similarity to human SRSF7 harbouring a single RRM and one zinc knuckle) was also enriched.

In the pulldown with *MIR398b-SL*, RS41 (RS subfamily), and the SR subfamily members SR34 and SR34a (orthologous to human SRSF1 proteins with an RRM and a pseudo-RRM), were identified as interactors. We further detected enrichment of the RSZ subfamily proteins RSZ21, RSZ22, and RSZ22a, and the plant-specific RS2Z32 protein (RS2Z subfamily; single RRM and two zinc knuckles). Among single-RRM SR proteins, SC35 (an SRSF2 orthologue) and the plant-specific SCL30 and SCL30a were enriched. The SR-like protein At-SR45 showed a log_2_ fold change of 0.7957. As it did not meet our significance criterion, it was not classified as an interactor.

### Impact of interactors on pri-miRNA and mature miRNA levels

**Fig. 3:**
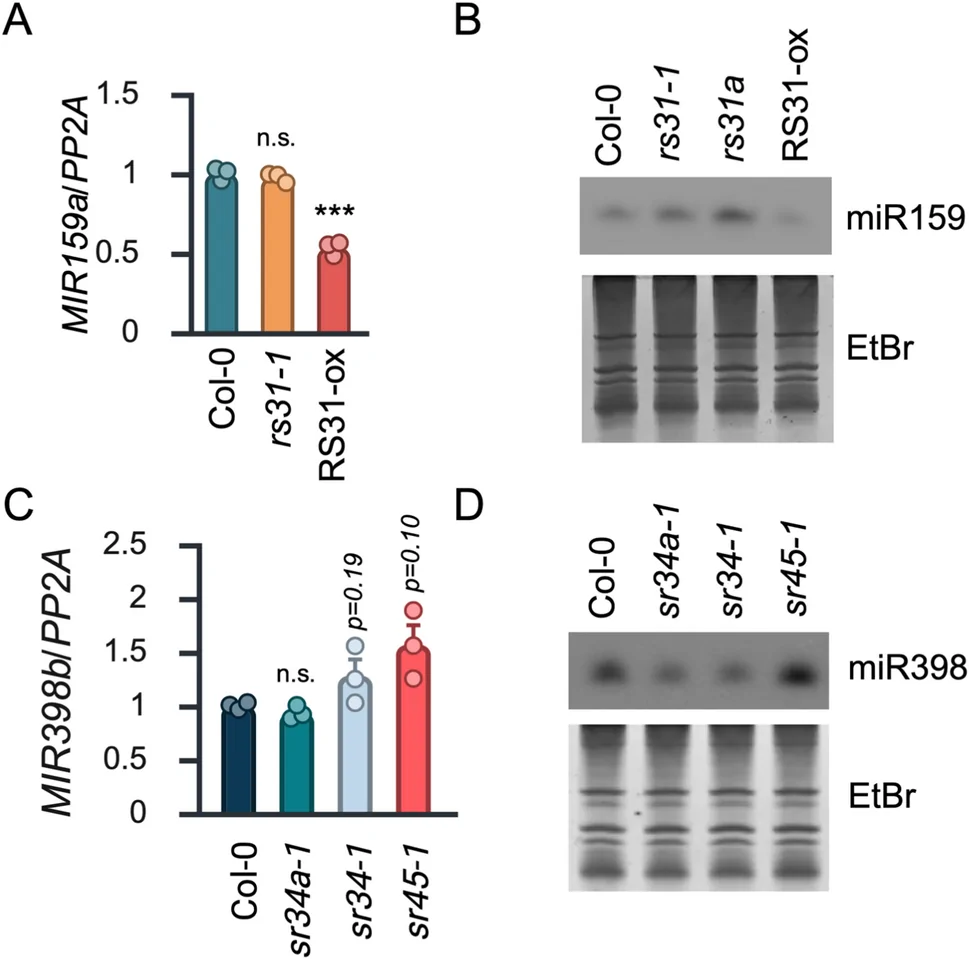
Impact of SR proteins on miR159 and miR398 levels. **A** RT-qPCR analysis of *MIR159a* levels in Col-0, *rs31-1*, *rs31a* and RS31-ox lines. **B** RNA gel blot analysis of miR159 in Col-0, *rs31-1*, *rs31a* and RS31-ox lines. Ethidium bromide staining of the gel serves as a loading control. **C** RT-qPCR analysis of *MIR398b* levels in Col-0, *sr34a-1*, *sr34-1*, and *sr45-1*. **D** RNA gel blot analysis of mature miR398 in Col-0, *sr34a-1*, *sr34-1*, and *sr45-1*. RT-qPCR values represent means from three independent biological samples, with error bars denoting SD. Significance was evaluated by Student’s t-test; *, **, and *** indicate *p* ≤ 0.05, *p* ≤ 0.01, and *p* ≤ 0.001, respectively, whereas n.s. denotes no significant difference.

To assess whether the identified *MIR159a-SL* and *MIR398b-SL* interactors influence miRNA biogenesis, we first analyzed pri-miRNAs and mature miRNA levels in selected SR protein mutants, as several SR proteins have recently been implicated in this process (Fig. 3).

For *MIR159a-SL*, RS31 was identified as an interactor. We analyzed the *rs31-1* mutant [37] and plants constitutively overexpressing RS31 (RS31-ox) [38]. As some peptides shared between the paralogs RS31 and RS31a and therefore cannot be unambiguously assigned by mass spectrometry, we also included the *rs31a* knockdown line carrying an artificial miRNA (amiR) construct against *RS31a* (amiR-31a-E2) [39]. *Pri-miR159a* levels were unchanged in *rs31-1* but significantly reduced in RS31-ox plants relative to wild type (Fig. 3A). By contrast, mature miR159 levels were significantly increased in the *rs31-1* mutant and the amiR *rs31a* knockdown line, whereas they were modestly but not significantly (*p* = 0.09) decreased in RS31-ox (Fig. 3B, Additional file 3, Fig S4A) (Additional file 4, Fig. S20: Uncropped gels and blots to Fig. 3B and Fig. S4A), These results suggest an inverse relationship between RS31/RS31a abundance and mature miR159 accumulation. Because the amiR-*rs31a* construct is based on the *pri-miR159a* backbone, the resulting high level of *pri-miR159a* signal precluded reliable quantification of the endogenous *pri-miR159a* levels in the amiR-*rs31a* knockdown line (Additional file 3, Fig. 4B) [39].

We also explored available transcriptome data from *rs31-1* mutant and RS31-ox plants for changes in *MIRNA* expression [38]. Because *MIRNAs* remain incompletely annotated, likely owing to their variable length and poorly defined transcript boundaries, we combined annotations from mirEX^2^ and Lepe-Soltero et al. to generate a custom annotation file for putative *MIRNAs* [53, 54] (Additional file 2, Table S4).

Using this annotation, we detected 326 putative *MIRNA* transcripts across the dataset. Of these, 207 were expressed at FPKM ≥ 1 in wild-type plants, 210 in *rs31-1*, and 199 in RS31-ox (Additional file 9, Table S12). Pairwise comparisons of FPKM values identified four transcripts with higher and two with lower abundance in *rs31-1* relative to wild-type at an unadjusted *p* ≤ 0.05. In RS31-ox, eight transcripts showed higher and 15 showed lower abundance at the same nominal threshold. As these comparisons were intended to generate hypotheses for further investigation, the reported counts describe exploratory candidates and do not imply significance after correction for multiple testing. Benjamini–Hochberg-adjusted *p*-values are also provided in Additional file 9, Table S12, allowing assessment of the evidence after accounting for multiplicity. Given the small number of nominally significant observations, these findings warrant cautious interpretation and further validation.

Unlike the RT-qPCR analysis, the RNA-seq reanalysis did not identify a change in *pri-miR159a* abundance meeting the nominal threshold in either *rs31-1* or RS31-ox. Sequence similarity within the *MIR159* family may complicate transcript quantification and contribute to the high variability of *MIR159a* FPKM values among the three wild-type replicates.

(Additional File 3, Fig. 3C, Additional file 9, Table S12). Next, we analyzed the level of *MIR398b* and mature miR398 in the respective mutants of the *MIR398b-SL* interactors SR34a and SR34. While the level of *pri-miR398b* was unchanged in both *sr34a-1* and *sr34-1* compared to the wild-type (Fig. 3C), the level of mature miR398 was moderately but significantly reduced in both mutants (Fig. 3D, Additional file 3, Fig. S5A) (Additional file 4, Fig. S21: Uncropped gels and blots to Fig. 3D and S5A).

The plant-specific RS subfamily protein RS41 was among the most strongly enriched *MIR398b-SL* interactors and showed one of the lowest *p*-values. It is known that RS41 and RS40 directly bind to a subset of miRNA precursors in vivo and act together with HIGH OSMOTIC GENE EXPRESSION 5 (HOS5) to facilitate the biogenesis of miRNAs [25]. To elucidate the effect of RS41 protein on miR398 expression, we employed the *rs40 rs41* double mutant owing to the significant homology and redundancy of both RS proteins as previously described [25]. Contrary to the unchanged level of *pri-miR398b* (Additional file 3, Fig. S5B), the level of mature miR398 was significantly reduced in the *rs40 rs41* double mutant compared to the wild-type (Additional file 3, Fig. S5C) (Additional file 4, Fig. S21: Uncropped gels and blots to Fig. S5C).

Together, these results indicate that distinct SR proteins, beyond the previously characterized RS40 and RS41, influence the accumulation of selected mature miRNAs, often without corresponding changes in the levels of their cognate pri-miRNAs.

To further assess the functional relevance of identified interactors of *MIRNA-SLs* we selected additional proteins containing classical RNA-binding domains for analysis. Among the interactors of *MIR398b-SL*, we identified the helicase BRR2a. In the *brr2a-2* (*cäö*) mutant, in which threonine at position 895 is substituted by isoleucine *pri-miR398b* and mature miR398 levels were decreased, suggesting that BRR2a affects *pri-miR398b* and mature miR398, in addition to its previously described effects on other miRNAs [66] (Additional file 3, Fig. S6A-C) (Additional file 4, Fig. S22: Uncropped gels and blots to Fig. S6A-C). Furthermore, in the *nuc1-2* mutant defective in a nucleolin-like protein (NUC1), levels of *pri-miR398b* and of mature miR398 were significantly reduced (Additional file 3, Fig. S6A-C) (Additional file 4, Fig. S22: Uncropped gels and blots to Fig. S6A-C).

Because miRNA levels vary widely in response to different environmental stimuli [66], we tested whether BRR2A or NUC1 affects *pri-miR398b* and mature miR398 accumulation after exposure to salt and abscisic acid (ABA), a key hormone in plant stress responses. For *brr2a-2* and *nuc1-2*, the levels of *pri-miR398b* were stronger reduced compared to wild-type upon exposure to 200 mM NaCl than under control conditions (Additional file 3, Fig. S6D) (Additional file 4, Fig. S22: Uncropped gels and blots to Fig. S6D). Mature miR398 levels were also strongly reduced in *brr2a-2* and *nuc1-2* under salt stress (Additional file 3, Fig. S6E/F) (Additional file 4, Fig. S22: Uncropped gels and blots to Fig. S6E/F). In response to ABA, *pri-miR398b* levels were modestly, but not significantly reduced to 50% in *brr2a-2* (*p* = 0.12) whereas mature miR398 levels were not affected (Additional file 3, Fig. S6G-I) (Additional file 4, Fig. S22: Uncropped gels and blots to Fig. S6G-I). Similarly, neither *pri-miR398b* nor mature miR398 differed significantly between *nuc1-2* and wild-type upon ABA treatment. These results suggest that pri-miRNA-associated proteins, including BRR2A and NUC1, can modulate miRNA expression in response to changing environmental conditions, such as salt stress.

### Identification of RS31 binding sites on *MIRNAs *in vivo

**Fig. 4:**
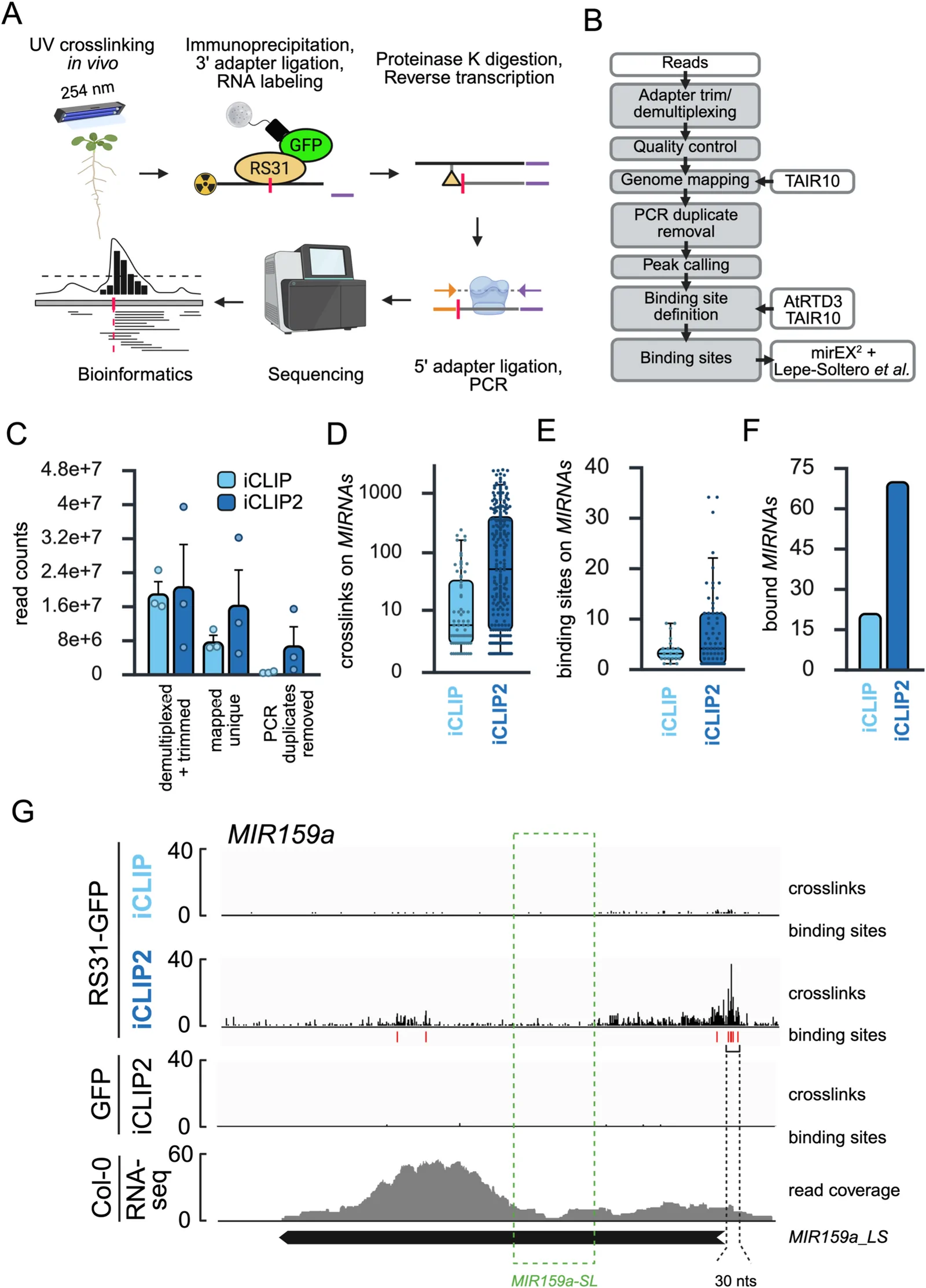
Plant iCLIP2 identifies RS31-GFP binding sites on *MIRNAs*. **A** Outline of the iCLIP2 procedure. **B** Bioinformatics workflow. **C** Number of reads after demultiplexing, adapter removal, trimming and quality control, mapped reads, uniquely mapped reads, and reads after deduplication. **D**,** E** Number of RS31-GFP crosslink and binding sites on *MIRNAs* in iCLIP and plant iCLIP2. **F** Number of RS31-GFP *MIRNA* targets in iCLIP and plant iCLIP2. **G** Crosslink sites and significant binding sites of RS31-GFP on *MIR159a* as determined by iCLIP and iCLIP2. For comparison, crosslink sites and significant binding sites of GFP are shown alongside a coverage profile from Col-0 wild-type plants. The Col-0 read coverage data were obtained from [38]

To investigate the interaction between *MIRNA* transcripts and SR proteins in vivo and to test whether RS31 directly contacts pri-miRNAs, we employed iCLIP-based approaches (Fig. 4A). First, we re-analyzed our previously published RS31 iCLIP data set [38]. To identify RS31 binding to pri-miRNAs, our custom annotation file for potential *MIRNAs* (Additional file 2, Table S4) was integrated in the bioinformatics pipeline (Fig. 4B). The coordinates of the identified binding sites were then compared with the *MIRNA* annotation file and candidate RS31 bound pri-miRNAs were extracted. In this workflow, crosslink sites were first identified at nucleotide resolution, and binding sites were subsequently defined computationally from the crosslink-site distribution according to the established pipeline.

However, this methodology produced only limited evidence for RS31 binding to pri-miRNAs, as relatively few crosslinks were detected and, consequently, binding sites were identified at only a small number of *MIRNA* loci (Additional file 10, Table S13). This is likely due, at least in part, to the low abundance of *MIRNA* transcripts in Arabidopsis [27, 32]. We therefore performed RS31 iCLIP using our recently established more sensitive plant iCLIP2 protocol [32]. Plant iCLIP2 retains the RS31-GFP immunopurification procedure used in the previous iCLIP protocol [38] while incorporating the substantially more efficient library preparation introduced by König and colleagues [69].

Briefly, plants expressing the RS31-GFP fusion protein under control of its native promoter in the *rs31-1* background were subjected to UV crosslinking. RS31-GFP and associated RNAs were precipitated from the lysates using GFP-Trap^®^ beads. RNAs were radioactively labeled, and RNA-protein complexes were separated by denaturing gel electrophoresis, blotted onto a nitrocellulose membrane and subjected to autoradiography for subsequent preparative isolation of RNA-protein complexes (Additional file 3, Fig. S7A) (Additional file 4, Fig. S23: Uncropped autoradiograms and gels to Fig. S7A). Representative RNase I controls demonstrating the presence of RNA co-immunoprecipitated with RS31-GFP have previously been published [38]. The indicated membrane regions were excised and subjected to library preparation for three biological replicates. Libraries were amplified by preparative PCR using nine cycles (Additional file, 3, Fig. S7B) (Additional file 4, Fig. S23: Uncropped autoradiograms and gels to Fig. S7B). As a control, we used transgenic plants expressing GFP alone under the control of the CaMV 35 S promoter (Additional file 3, Fig. S7C/D) (Additional file 4, Fig. S24: Uncropped autoradiograms and gels to Fig. S7C/D). After sequencing, reads were processed according to our bioinformatics workflow supplemented with the *MIRNA* annotation file (Fig. 4B) [32].

Direct comparison between the previous iCLIP dataset with the plant iCLIP2 dataset showed a substantially higher average number of uniquely mapped reads after processing the raw reads. After removal of the PCR duplicates generated during library amplification, plant iCLIP2 retained an average of around 10 million reads, compared with 0.4 million reads in iCLIP (Fig. 4C, Additional file 10, Table S13). This was accompanied by almost one order of magnitude increase in the number of crosslink sites detected by plant iCLIP2 compared with iCLIP (Fig. 4D, Additional file 10, Table S14). Consequently, more binding sites on pri-miRNAs were identified using plant iCLIP2 (Fig. 4E, Additional file 10, Table S14 and S15). In total, our modified plant iCLIP2 pipeline identified 68 candidate RS31 pri-miRNA targets compared with 21 in the previous iCLIP dataset (Fig. 4F, Additional file 10, Table S14 and S15).

iCLIP has been shown to be biased to highly expressed transcripts [27]. To test whether we can also find binding sites on pri-miRNAs with low expression levels using our plant iCLIP2 protocol, we monitored expression levels of pri-miRNAs in the RNA-seq data of wild-type plants and compared those with the expression levels of bound pri-miRNAs [38]. Pri-miRNAs detected in iCLIP and plant iCLIP2 displayed similar high median levels, although in plant iCLIP2 more pri-miRNAs with lower expression levels were also detected (Additional file 3, Fig. S8). Overall, our improved plant iCLIP2 protocol enables more efficient identification of RBP-binding sites on short-lived pri-miRNAs than iCLIP.

As proof-of-concept, we checked whether *pri-miR159a* is among the plant iCLIP2 targets and thus directly bound by RS31 in vivo*.* The direct comparison between iCLIP and plant iCLIP2 displayed an increased number of *MIR159a* crosslink sites in our improved plant iCLIP2 data set (Fig. 4G). In contrast to iCLIP, three RS31 binding sites were identified on the annotated *MIR159a* using plant iCLIP2, confirming *pri-miR159a* as a direct target of RS31. Surprisingly, no binding sites could be called within the *MIR159s-SL* region, instead two binding sites were identified upstream and one downstream of the stem-loop. Nevertheless, individual crosslink sites were identified within the stem-loop region, although they did not meet the criteria for binding-site calling in our pipeline. This suggests that RS31 may contact the *pri-miR159a* stem-loop transiently or at low frequency in vivo, which could explain why RS31 co-purified with the *MIR159a-SL* in the in vitro RNA affinity pulldown. In addition, we observed strong enrichment of crosslink sites and four RS31 binding sites in a region just 30 nucleotides upstream of the annotated *MIR159a* sequence. This suggests that the current annotation of *MIR159a* may be incomplete at the 5’ end, an interpretation supported by RNA-seq data from Col-0 wild-type plants (Fig. 4G) [38]. Thus, RS31 appears to bind most prominently upstream of the annotated *MIR159a* stem-loop, while also making weaker or transient contacts within the stem-loop region.

Next, we analyzed the sequence characteristics of the identified binding sites. Given the limited number of binding sites on *MIRNAs*, we performed motif discovery using all transcriptome-wide binding sites to obtain a more robust result (Additional file 11, Table S16). Consistent with earlier findings indicating that RS31 preferentially binds to CU-rich motifs, STREME identified a similar CU-rich motif in the plant iCLIP2 datasets derived from RS31-GFP plants (Additional file 3, Fig. S9A) [69]. A parallel search for k-mer motifs (trimers) enriched within the binding sites confirmed enrichment of CU-rich sequence combinations (Additional file 3, Fig. S9B). The presence of CU-rich motifs within the *MIR159a-SL* is consistent with the possibility that RS31 transiently contacts the *MIR159a* stem-loop region, which may explain its co-purification in the in vitro RNA-affinity pulldown (Additional file 3, Fig. S9C).

Cross-referencing differentially abundant *MIRNAs* with the plant iCLIP2 data showed that two RS31-bound pri-miRNAs were moderately, but significantly, up-regulated in *rs31-1 vs.* wild-type (Additional file 3, Fig. S10A). Conversely, six RS31-bound pri-miRNAs were significantly down-regulated and three bound pri-miRNAs were up-regulated in RS31-ox compared with wild-type (Additional file 3, Fig. S10 B). Among these, *pri-miR156c* showed reciprocal changes in *rs31-1* and RS31-ox. A closer look showed nine consecutive RS31 binding sites upstream of the *MIR156c* stem-loop at the 5’ end of the annotated precursor (Additional file 3, Fig. S10C). In addition, there are eight closely spaced binding sites in region of the 3’ base of the annotated stem-loop.

Interestingly, *pri-miR398c* was among the RS31-bound pri-miRNA transcripts that were significantly down-regulated transcripts in RS31-ox (Additional file 3, Fig. S10B). Like *pri-miR398c*, *pri-miR398b* was also down-regulated in RS31-ox, although not significantly (*p* = 0.07). The annotated *MIR398b* and *MIR398c* stem-loop sequences are almost identical. However, RS31 was not co-purifed in the *MIR398b-SL* affinity pull-down. Closer examination of the RS31-binding site distribution on *pri-miR398c* (Additional file 3, Fig. S10 D) and *pri-miR398b* (Additional file 3, Fig. S11A) revealed that binding sites were exclusively localized down-stream of the annotated stem-loops and there were no CU-rich RS31-binding motifs within the annotated *pri-miR398c* and *pri-miR398b* stem-loops. Notably, many additional binding sites were located downstream of the annotated *MIR398c* and *MIR398b* regions, suggesting that annotations of these pri-miRNA transcripts may be incomplete. Three consecutive binding sites were located close to the base of the *pri-miR398b* stem-loop (Additional file 3, Fig. S11A/B). According to the short base-to-loop processing mechanism, this region is important for proper processing of *pri-miR398b* by DCL1 [21]. An imperfectly paired lower stem of ~ 15 nucleotides below the miRNA/miRNA* duplex, followed by an internal loop, determines the initial DCL1 pri-miRNA cleavage site. Subsequent cleavage of the pre-miRNA at a distance of ~ 21 nucleotides from the first cut then releases the miRNA/miRNA* duplex. To test whether altered RS31 abundance affects mature miR398 accumulation, we performed small RNA gel blots. Mature miR398 levels showed reciprocal but no significant trend, with increased levels in *rs31-1* and reduced levels in RS31-ox (Additional file 3, Fig. S11C) (Additional file 4, Fig. S26: Uncropped gels and blots to Fig. S11C). We further validated the significant decrease of *pri-miR398c* in RS31-ox and the absence of significant change in the *rs31-1* mutant by RT-qPCR (Additional file 3, Fig. S11D). In accordance with the RNA-seq data, *pri-miR398b* levels were reduced in RS31-ox, albeit not significantly (*p* = 0.07) (Additional file 3, Fig. S11D).

Together, these data show that plant iCLIP2 significantly improves the in vivo detection of crosslink sites and binding sites on pri-miRNA transcripts in vivo and enables identification of a substantially broader set of *MIRNA* transcript targets than the previous iCLIP protocol.

### Identification of SR34a binding sites on *MIRNAs in vivo*

**Fig. 5:**
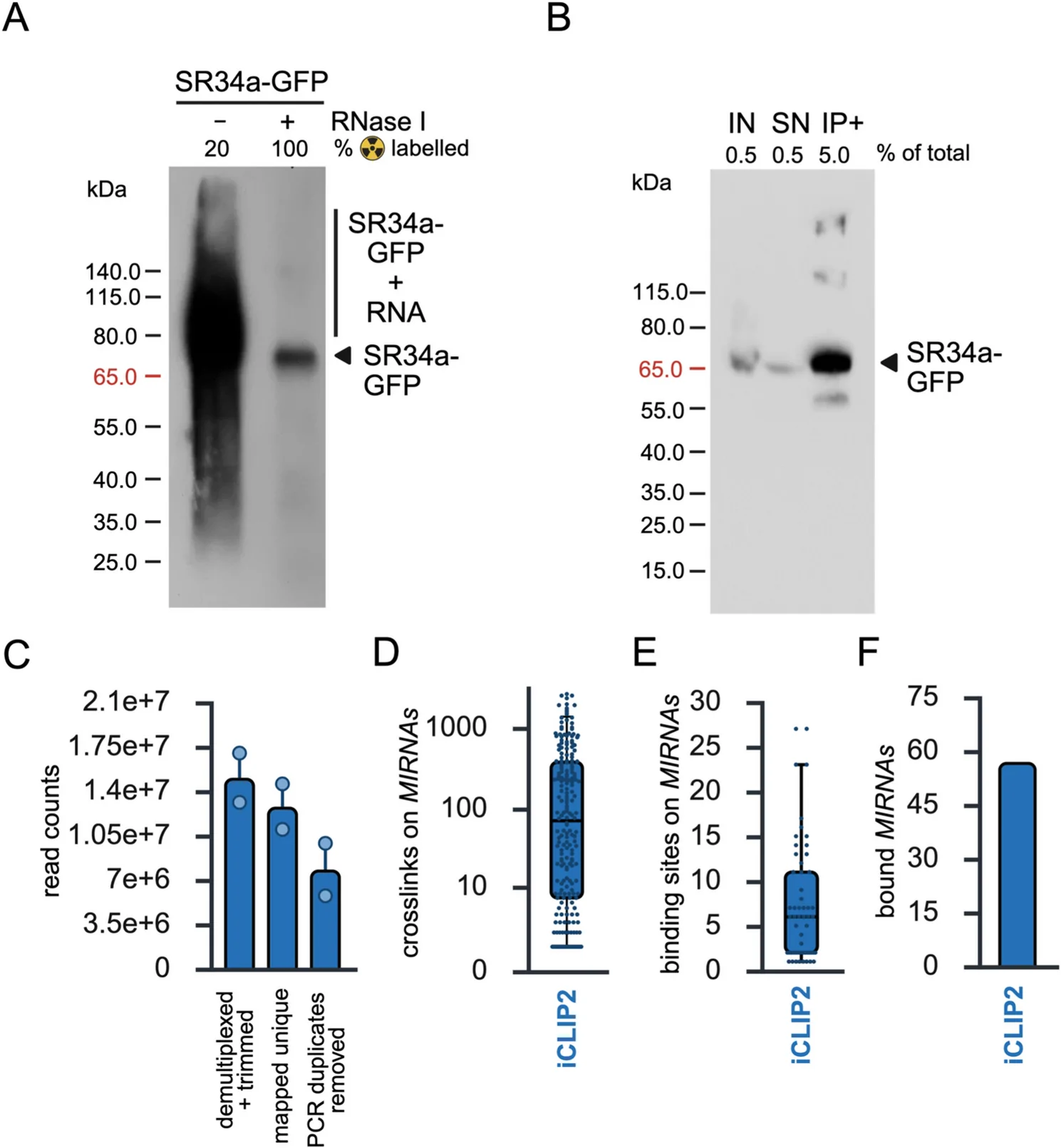
Plant iCLIP2 of SR34a-GFP. **A** Autoradiogram of RNA-protein complexes from SR34a::SR34a-GFP *sr34a-1* plants after UV crosslinking and immunoprecipitation with GFP-Trap^®^ beads. Samples qwere treated with RNase I as indicated. The radioactive smear above the expected size of SR34a-GFP corresponds to SR34a-GFP crosslinked to RNA. The arrowhead marks the expected position of SR34a-GFP after RNase treatment. **B** Immunoblot analysis of SR34a::SR34a-GFP *sr34a* plants. Aliquots of the input fraction (IN, 0.5%), supernatant (SN, 0.5%) and the immunoprecipitated fraction (IP+, 5%) were separated by NuPAGE and probed with the α-GFP antibody. **C** Number of reads retained after demultiplexing, adapter removal, trimming and quality control, mapped reads, uniquely mapped reads, and reads after removal of PCR duplicates. **D**, **E** Number of SR34a-GFP crosslink sites (**D**) and binding sites (**E**) identified on *MIRNAs* by iCLIP and plant iCLIP2. **F** Number of SR34a-GFP *MIRNA* targets identified by plant iCLIP2.

To identify in vivo binding of SR34a to pri-miRNAs, we performed plant iCLIP2 on transgenic plants expressing SR34a-GFP under control of its native promoter in the *sr34a-1* mutant background. This line had previously been used to determine the transcriptome-wide binding landscape of SR34a in germinating seeds [34]. Plants were maintained for two weeks at 20 °C under alternating 12-h light and 12-h dark periods before UV irradiation. SR34a-GFP and associated RNAs were immunoprecipitated from crude lysates using GFP-Trap^®^ beads followed by radioactive labeling and denaturing gel electrophoresis (Fig. 5A) (Additional file 4, Fig. S25: Uncropped autoradiograms and gels to Fig. 5A). The radioactive smear above the expected size of the SR34a-GFP fusion protein, which was strongly reduced after treatment with a high concentration RNase I, indicates efficient RNA-protein crosslinking and recovery of SR34a-GFP-associated RNAs.

**Fig. 6:**
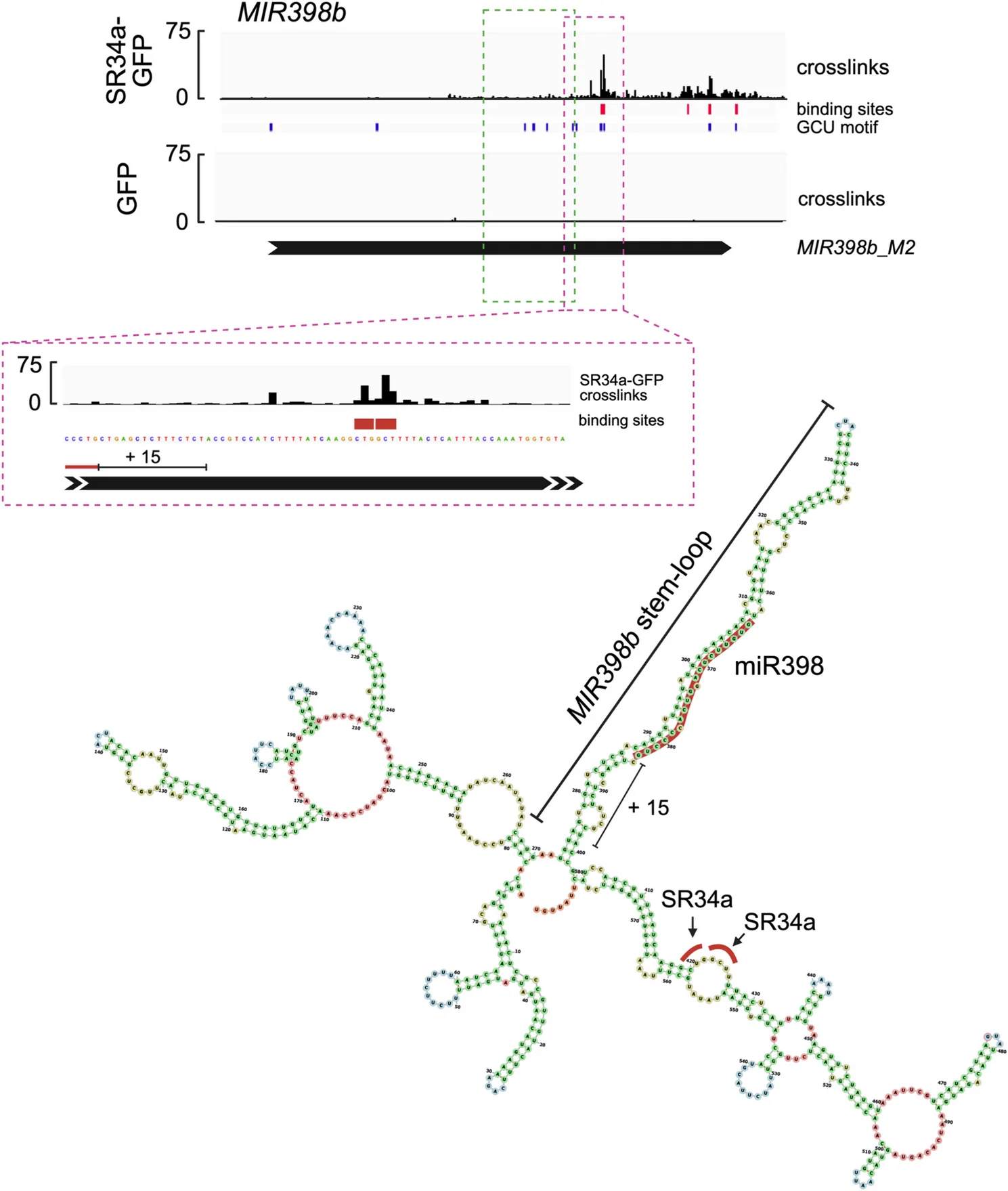
SR34a-GFP binding sites on *pri-miR398b.*Top: Crosslink sites and significant SR34a-GFP binding sites on *MIR398b* as determined by plant iCLIP2. The GFP control track is shown for comparison. Red boxes mark identified binding sites, and blue boxes indicate GCU motifs within the annotated *MIR398b_M2* region shown as a black arrow. The green dashed box marks the region of the annotated *MIR398b* stem-loop region. The magenta dashed box highlights SR34a-GFP binding sites downstream of the base of the *MIR398b* stem-loop region. Bottom: In silico predicted secondary structure of *MIR398b*. Red bars indicate the binding sites near the base of the stem-loop. The + 15 marks the region important for correct processing of *pri-miR398b*.

Plant iCLIP2 statistics are shown in Fig. 5C-F and Additional file 12, Table S17. Overall, the number of reads retained after processing and removal of PCR duplicates was in a range similar range to that obtained for RS31-GFP. This led to the identification of a total of 65,775 crosslink sites (Fig.5D, Additional file 12, Table S18) and 412 binding sites (Fig. 5E, Additional file 12, Table S18) distributed across *57* pri-miRNAs bound by SR34a-GFP (Fig. 5F, Additional file 12, Table S19). Motif discovery using STREME and k-mer enrichment confirmed the predominant GCU motif previously identified for SR34a-GFP in germinating seeds (Additional file 3, Fig. S13A, B) [34]. Because SR34a co-purified with the *MIR398b-SL* in the affinity pulldown, we first checked whether SR34a-GFP also binds to the *pri-miR398b *in vivo. Indeed, a total of four binding sites were identified on the annotated *MIR398b* region, all located downstream of the annotated stem-loop (Fig. 6). An additional binding site was found just four nucleotides downstream of the annotated *MIR398b* region. A similar binding pattern with a binding site located downstream of the stem-loop and outside the annotated precursor region, was observed for *MIR398c* (Additional file 3, Fig. S14A). Importantly, although mature miR398 levels were significantly reduced in *sr34a-1* (Fig. 3D, Additional file 3, Fig. S5A) (Additional file 4, Fig. S21: Uncropped gels and blots to Fig. 3D and S5A), the level of *pri-miR398b* and *pri-miR398c* levels did not differ significantly between *sr34a-1* and wild-type (Fig. 3C, Additional file 3, Fig. S14B).

### Comparative analysis of RS31-GFP and SR34a-GFP *MIRNA* targets

Comparison of the pri-miRNAs directly bound by RS31-GFP and SR34a-GFP revealed 48 common pri-miRNA targets for the two SR proteins, corresponding to 70.6% of RS31-GFP targets and 84.2% of SR34a-GFP targets (Additional file 3, Fig. S15). This suggests that RS31 and SR34a associate with a largely shared subset of pri-miRNA transcripts. In contrast, 20 pri-miRNAs were identified exclusively as RS31-GFP targets and nine pri-miRNAs as SR34a-GFP targets, corresponding to 29.4% and 15.7% of their respective target sites.

Moro and colleagues have described loop-to-base (LTB) and base-to-loop (BTL) processing modes for more than one hundred evolutionarily conserved miRNA precursors. About one third of these precursors are processed in the LTB or sequential (s)LTB mode (i.e., *MIR779*, *MIR159* and *MIR319* families). Among pri-miRNAs processed in the BTL direction, members of the *MIR169* family and *MIR402* are processed in the sequential (s)BTL mode. The predominant processing mode was known for 31 of the 77 pri-miRNA transcripts identified as targets of either RS31-GFP or SR34a-GFP (Additional file 3, Fig. S15). Notably, neither RS31 nor SR34a showed an obvious preference for binding pri-miRNAs of a specific processing type, as all processing types are represented in expected proportions.

Next, we asked whether RS31 and SR34a preferentially bind specific regions of the miRNA precursors. For this analysis, the annotated *MIRNAs* were divided into regions upstream and downstream of the stem-loop, the stem-loop itself, and a base region defined as 25 nucleotides upstream and downstream of the stem-loop base (Additional file 3, Fig. S16A). Because the number of crosslinks and consequently the number of identified binding sites can correlate with the pri-miRNA abundance and may not directly reflect the binding affinity, we scored whether at least one binding site was present in each region rather than counting the total number of binding sites per region. Thus, individual pri-miRNAs could be assigned to more than one region. As previously described, RS31 binding was enriched towards the 5’ region of transcripts [38]. Consistently, RS31 binding was detected upstream of the stem-loop for 46 of 68 RS31-bound pri-miRNAs, downstream of the stem-loop for 25, within the stem-loop for 15, and at the base of the stem-loop for 10 (Additional file 3, Fig. S16B). A separate analysis of pri-miRNAs processed by BTL or LTB mechanisms showed a comparable distribution, although the number of pri-miRNAs in these subsets was lower (Additional file 3, Fig. S16B). A similar regional distribution was observed for SR34a (Additional file 3, Fig. S16B). Thus, despite belonging to different SR protein subfamilies, RS31 and SR34a showed broadly similar regional binding distributions on pri-miRNAs. For both RS31 and SR34a binding was detected most frequently upstream of the stem-loop, suggesting that these SR proteins preferentially associate with flanking regions of pri-miRNA transcripts rather than with the stem-loop itself, regardless of processing type. However, no clearly defined general pattern of pri-miRNA binding was apparent.

Among the common evolutionarily conserved pri-miRNA targets that are processed in the BTL direction was *pri-miR167a*. In a region approximately 500–1000 nucleotides downstream of the annotated *MIR167a* stem-loop, one RS31 and seven SR34a binding sites were detected (Additional file 3, Fig. S17A). In addition, four RS31 and five SR34a binding sites were located in an approximately 40-nucleotide CU-rich region only 15 nucleotides upstream of the base of the stem-loop. Strikingly, RS31 and SR34a binding sites partially overlapped in this region.

In contrast, SR34a and RS31 showed distinct binding patterns on the BTL- processed *pri-miR171b* and *pri-miR396b* (Additional file 3, Fig. S17B, C). For *pri-miR171b*, only SR34a showed a significant binding site at the base of the stem-loop, whereas significant RS31 binding sites were located mainly at the 5’ end of the annotated precursor. Conversely, for pri-*miR396b*, significant binding sites in the stem-loop base region were detected only for RS31.

Overall, RS31 and SR34a showed a substantially overlapping set of pri-miRNA targets, indicating that these SR proteins associate with a largely shared subset of pri-miRNA transcripts. However, neither protein showed an obvious preference for pri-miRNAs with a specific processing mode. Instead, binding by both proteins was detected most frequently upstream of the stem-loop, whereas binding within the stem-loop or at the stem-loop base was less common. Individual examples further showed that RS31 and SR34a can bind either overlapping or distinct regions of the same precursor. Thus, RS31 and SR34a appear to associate preferentially with pri-miRNA flanking regions, but their precise binding patterns are target-specific.

## Discussion

MiRNAs are essential post-transcriptional regulators that modulate gene expression by affecting mRNA stability and translation. By targeting transcription factors and other regulatory proteins, miRNAs can contribute to fine-tuned rather than binary regulation, thereby supporting robustness and adaptability in gene expression. In this context, RBPs involved in miRNA processing are likely to add specificity and regulatory versatility to miRNA production.

Here, we established a biotin-based pulldown strategy to identify candidate RBPs interacting with pri-miRNA SLs. The stem-loops of *pri-miR159a* processed from loop-to-base and *pri-miR398b* processed from base-to-loop were selected as representative models.

Using this approach, we identified 110 significantly enriched proteins for *MIR159a-SL* and 235 for *MIR398b-SL* relative to an empty bead control, which we define as candidate interactors. The suitability of the approach is supported by the recovery of pri-miRNA processing components and RBPs previously implicated in pri-miRNA processing. In addition, the presence of several identified proteins in current Arabidopsis RNA interactomes is consistent with RNA association in vivo. Nevertheless, enrichment in pulldown assays should be interpreted cautiously, as it does not by itself establish direct regulatory activity in vivo. Notably, the number of interactors identified for *MIR398b-SL* was considerably higher than in our previous pulldown using the cleavage-deficient CRISPR/Cas nuclease Cys4* [35].

Among the enriched proteins, several RRM domain-containing proteins belonged to the SR-rich protein family. Building on the established roles of Arabidopsis RS40 and RS41 in pri-miRNA binding and processing [25], and evidence linking mammalian SR proteins to miRNA biogenesis [70–72], we first examined whether RS31 and its close paralog RS31a influence the abundance of *pri-miR159a* and the corresponding mature miR159. Mature miR159 levels were significantly increased in the *rs31-1* mutant and the *rs31a* knockdown line, whereas RS31-ox plants showed a modest, non-significant decrease (*p* = 0.09). These observations suggest an association between altered RS31 and RS31a abundance and mature miR159 accumulation, but do not establish direct regulation of *pri-miR159a* processing. *Pri-miR159a* levels were unchanged in *rs31-1* but significantly reduced in RS31-ox, indicating that precursor and mature miRNA abundance do not respond uniformly to altered RS31 expression. Exploratory transcriptome analyses identified additional pri-miRNAs with nominal abundance differences upon RS31 misexpression, providing candidates for further validation rather than evidence for a broad regulatory role.

A related pattern emerged for the SR subfamily proteins SR34a and SR34, both of which were purified with *MIR398b-SL*. SR34a and SR34 are homologues of the animal SR protein SRSF1, which modulates the processing of specific miRNAs in mammalian cells [71]. Although *pri-miR398b* levels were unchanged in *sr34a-1* and *sr34-1*, mature miR398 levels were slightly reduced in both mutants. This observation suggests that SR34a and SR34 may contribute to miR398 accumulation, although the modest effect size warrants a cautious interpretation.

By contrast, no significant differences in steady-state levels of either *pri-miR398b* or mature miR398 were detected in *sr45-1* mutants, which is consistent with the non-significant enrichment of SR45 protein in the pulldown. However, previous RIP-seq experiments have shown that SR45 binds to *pri-miR398c *in vivo [68]. Thus, a regulatory influence of SR45 on miR398 expression cannot be excluded, particularly if such an effect is context-dependent or specific to particular *MIR398* family members.

Previous work showed that RS40 and RS41 affect the processing of a subset of pri-miRNAs through direct binding [25]. Although miR398 was not previously identified as differentially regulated by RS40 and RS41, our data suggest that mature miR398 expression is altered in *rs40 rs41* mutants. This discrepancy may reflect differences in growth conditions or harvest time, especially because miR398 expression is diurnally regulated and reaches maximum expression at dusk [73].

Beyond SR proteins, the RNA helicase BRR2a was also recovered in the *MIR398b-SL* pulldown. The significantly reduced *pri-miR398b* and miR398 levels observed in *brr2a-2* mutants are consistent with previous evidence that BRR2a modulates pri-miRNA secondary structure to improve the efficiency and fidelity of processing from pri-miRNA to mature miRNA [66]. Loss of BRR2a function may therefore impair the structural suitability of multiple pri-miRNAs and reduce miRNA accumulation.

NUC1, a nucleolin-like nucleolar protein, promotes early pre-rRNA processing and ribosome biogenesis in plants. Loss of NUC1 in *nuc1-2* mutants alters rRNA precursor accumulation and causes nucleolar disorganization accompanied by developmental defects [74]. The co-purification of NUC1 with *MIR398b-SL*, together with the significantly reduced levels of *pri-miR398b* and mature miR398 in *nuc1-2* mutants, suggests that NUC1 may also contribute to miR398 biogenesis. The reduction in miR398 levels in *brr2a-2* and *nuc1-2* mutants was more pronounced under salt stress than under regular growth conditions. This finding suggests that regulatory effects that appear weak or difficult to detect under standard conditions may become more evident when plants experience specific environmental stimuli.

Across several RBP mutants, the observed differences in steady-state pri-miRNA or mature miRNA abundance were relatively small, as has also been reported in previous studies [17, 24, 25]. Such modest changes are not unexpected for miRNAs, which often act as modulators of gene expression. Moreover, analyses of whole plant tissue may obscure effects that occur only in specific cell types, and functional redundancy among related proteins may further limit the detection of changes in individual mutants, as observed for RS40 and RS41 [25]. These considerations should be taken into account when interpreting the magnitude of the observed effects.

Pulldown-based approaches have also been used in other organisms to identify RBPs associated with miRNA precursors. In mammals, for example, a global inventory of RBPs interacting with 72 pre-miRNA hairpins was assembled using pulldown with biotinylated antisense oligonucleotides across eleven cell lines, leading to the identification of 180 RBPs implicated in miRNA biogenesis [75]. Such inventories provide valuable candidate regulators, but they do not resolve the precise binding sites through which individual RBPs may influence processing.

To improve the detection of RBP binding sites on lowly expressed *MIRNAs*, we modified the iCLIP library strategy and developed an optimized plant iCLIP2 workflow. Overall, these optimizations improved the efficiency of library preparation, as indicated by the increased number of uniquely mapped reads after removal of PCR duplicates. To identify RBP binding on poorly annotated *MIRNAs*, we also established a new *MIRNA* annotation file and combined it with the optimized plant iCLIP2 workflow. This strategy enabled the identification of more reproducible binding sites corresponding to RBP interaction sites on pri-miRNA transcripts in vivo. However, despite the improved library preparation efficiency, detection remained biased toward relatively highly expressed *MIRNAs*. Consequently, *MIRNA* binding targets are likely still underestimated.

Using this approach, we identified 68 pri-miRNA targets for RS31-GFP and 57 pri-miRNA targets for SR34a-GFP. Among these targets, significant binding sites were detected on *pri-miR159a* for RS31-GFP and on *pri-miR398b* for SR34a-GFP. These findings demonstrate direct in vivo binding of RS31 to *pri-miR159a* and SR34a to *pri-miR398b*, validating selected interactions identified by the in vitro pulldown. Together with the abundance changes observed in the corresponding mutant lines, they support prioritizing these proteins as candidate regulators of miRNA abundance. However, the binding and abundance data do not establish that the mapped interactions directly regulate precursor processing. Our study therefore extends the evidence for pri-miRNA binding to additional SR proteins beyond RS40 and RS41 [25] and provides nucleotide-resolution maps of their contacts in vivo.

Further analysis of RS31-GFP and SR34a-GFP showed that some identified binding sites are located in sequence regions important for processing pri-miRNAs into mature miRNAs, including sequence and structural features proximal to the miRNA/miRNA* duplex [21]. In base-to-loop processed pri-miRNAs, the miRNA/miRNA* duplex contains a lower stem of approximately 15 nucleotides followed by a substantial bulge, and these lower-stem features are important for accurate processing. Members of the *MIR156* and *MIR398* families in particular exhibited RS31-GFP binding sites close to the lower stem of the respective pri-miRNAs and showed differential expression in RS31 gain- and loss-of-function mutants. Together, these observations are consistent with a model in which selected RBPs bind near processing-relevant structural regions and thereby contribute to the regulation of specific miRNA precursors.

The binding-site analyses further indicate that RS31- and SR34a-associated sites are not confined to stem-loop structures but are also prominent in upstream and downstream flanking sequences. These findings highlight the importance of extended *MIRNA* annotations when analyzing RBP binding to miRNA precursors. Consistent with this, Li et al. (2020) used RACE to confirm that *pri-miR398b* and *pri-miR398c* transcripts extend beyond the boundaries annotated in mirEX^2^ [53, 76]. The preferential detection of RS31 and SR34a binding in flanking regions further suggests that these sequences may provide regulatory platforms that influence productive engagement of the adjacent hairpin by the processing machinery. A precedent is provided by FCA, which binds sequences flanking the *pri-miR172b* stem-loop and promotes its processing [77]. One hypothesis for future testing is that SR protein binding in flanking regions modulates hairpin accessibility by influencing local RNA conformation or the assembly of processing-associated ribonucleoprotein complexes. The outcome may depend on binding-site position, precursor architecture and the availability of other factors, potentially explaining both substrate selectivity and the different effects of individual SR proteins. The largely unchanged abundance of the corresponding pri-miRNAs does not exclude this possibility, because steady-state precursor measurements do not resolve processing flux, cleavage accuracy or the fraction of transcripts competent for processing. Nevertheless, altered mature miRNA stability, indirect regulatory effects and contributions from other *MIRNA* family members remain alternative explanations, and the functional relevance of individual flanking-region binding events requires experimental validation. Finally, the comparatively limited detection of binding sites within paired stem regions should be interpreted cautiously, as UV crosslinking is generally less efficient in double-stranded RNA regions. Reduced recovery of plant iCLIP2 signals in these regions therefore does not necessarily exclude interactions with such structures.

A large fraction of pri-miRNA targets was shared between RS31 and SR34a, with 48 common targets corresponding to 70.6% of RS31-bound and 84.2% of SR34a-bound pri-miRNAs. This extensive overlap raises the possibility that RS31 and SR34a contribute to combinatorial or partially redundant regulation of common miRNA precursors. The occurrence of overlapping or closely positioned binding sites is also consistent with the possibility of competitive binding at selected regions. Nevertheless, whether such competition occurs in vivo and whether it has a relevant effect on miRNA biogenesis remains unresolved. Addressing this question will require higher-order mutant combinations and targeted functional analyses that can distinguish individual, redundant, and potentially competitive contributions of these RBPs.

Taken together, our findings support a potential role for SR proteins in pri-miRNA processing, but their interpretation must also account for the established functions of these proteins in splicing regulation. Perturbation of SR proteins can alter transcriptome-wide alternative splicing and thereby indirectly influence miRNA biogenesis. However, transcriptome analyses of *rs31-1* and RS31-ox plants revealed neither differential expression nor differential alternative splicing of genes encoding core components of the miRNA processing machinery [38]. This observation argues against indirect effects mediated by changes in the expression or splicing of these core components, although contributions from other regulatory factors cannot be excluded. Conversely, many RS31 binding events have not been linked to detectable changes in alternative splicing, and previous iCLIP analyses revealed prominent binding within 5′ UTRs, whose functional significance remains unresolved [38]. These observations are consistent with additional functions of RS31 in RNA metabolism beyond splicing. Nevertheless, direct binding to pri-miRNAs does not establish a causal effect on their processing. Distinguishing the functional contributions of individual binding events from indirect effects and resolving the interplay among SR proteins will therefore be essential for defining their mechanistic roles in miRNA biogenesis.

## Conclusion

This study combines biotin-based pri-miRNA stem-loop pulldown with optimized plant iCLIP2 to expand the repertoire of proteins associated with plant miRNA precursors and resolve direct contacts of RS31 and SR34a in vivo. Binding-site mapping reveals interactions within stem-loops and their flanking regions, highlighting the importance of considering the broader precursor context. Together with changes in pri-miRNA and/or mature miRNA abundance in selected mutant and overexpression lines, these findings identify additional SR proteins as candidate regulators of miRNA abundance. Although the functional effects were generally modest and direct regulation of precursor processing remains to be established, the integration of protein discovery, in vivo binding maps and functional analyses provides a foundation for uncovering how SR proteins contribute to plant miRNA biogenesis.

## Supplementary Information

Below is the link to the electronic supplementary material.

Supplementary Material 1

Supplementary Material 2

Supplementary Material 3

Supplementary Material 4

Supplementary Material 5

Supplementary Material 6

Supplementary Material 7

Supplementary Material 8

Supplementary Material 9

Supplementary Material 10

Supplementary Material 11

Supplementary Material 12

## Data Availability

Plant iCLIP2 reads have been submitted to Sequence Read Archive (SRA) under the BioProject accession No.: PRJNA1478861. The mass spectrometry proteomics data have been deposited to the ProteomeXchange Consortium via the PRIDE partner repository with the dataset identifier PXD085168.

## Abbreviations

GO: Gene ontology
iCLIP: individual-nucleotide resolution crosslinking and immunoprecipitation
MIRNA: pri-miRNA
miRNA: microRNA
MS: mass spectrometry
nt: nucleotide
RBP: RNA-binding protein
SR: serine-arginine rich
